# Opportunities and obstacles in microbial synthesis of metal nanoparticles

**DOI:** 10.1111/1751-7915.14254

**Published:** 2023-03-25

**Authors:** Manuel Carmona, Ignacio Poblete‐Castro, Mahendra Rai, Raymond J. Turner

**Affiliations:** ^1^ Centro de Investigaciones Biológicas Margarita Salas‐CSIC Madrid Spain; ^2^ Universidad de Santiago de Chile Santiago Chile; ^3^ Sant Gadge Baba Amravati University Amravati Maharashtra India; ^4^ University of Calgary Calgary Alberta Canada

## Abstract

Metallic nanoparticles (MeNPs) are widely used in many areas such as biomedicine, packaging, cosmetics, colourants, agriculture, antimicrobial agents, cleaning products, as components of electronic devices and nutritional supplements. In addition, some MeNPs exhibit quantum properties, making them suitable materials in the photonics, electronic and energy industries. Through the lens of technology, microbes can be considered nanofactories capable of producing enzymes, metabolites and capping materials involved in the synthesis, assembly and stabilization of MeNPs. This bioprocess is considered more ecofriendly and less energy intensive than the current chemical synthesis routes. However, microbial synthesis of MeNPs as an alternative method to the chemical synthesis of nanomaterials still faces some challenges that need to be solved. Some of these challenges are described in this Editorial.

## INTRODUCTION

Richard Feynman gave in 1959 a lecture entitled ‘There's plenty of room at the bottom’. The introductory words of the talk were ‘I would like to describe a field, in which little has been done, but in which an enormous amount can be done in principle. What I want to talk about is the problem of manipulating and controlling things on a small scale’ (Feynman, 1959). This lecture anticipates the theoretical bases of nanotechnology. At present we know the importance of nanomaterials because they display singular physicochemical properties, such as optical, electronic or magnetic features, that make them highly appreciated in the industry for the development of a vast number of applications: delivery of drugs, antimicrobial therapies, tumour detection, photoimaging, photothermal therapy, cosmetic design, batteries and so on (Chopra et al., 2022; Pereira et al., 2015).

The well‐known and widely used physicochemical strategies for the synthesis of metallic nanoparticles (MeNPs) cause, however, negative environmental impact due to the use of pollutants and/or high demand for energy. Conversely, the biological production of MeNPs is an environmentally friendly and low energy consumption alternative that can render MeNPs of well‐defined size and shape. Microorganisms, fungi and plants have been demonstrated as good MeNPs producers. Among all these organisms, bacteria are the best choice because they offer the highest speed of production and the possibility of easy genetic modification (Singh et al., 2016).

## SEARCHING FOR THE BACTERIAL CHASSIS FOR MENPS PRODUCTION

**FIGURE 1:**
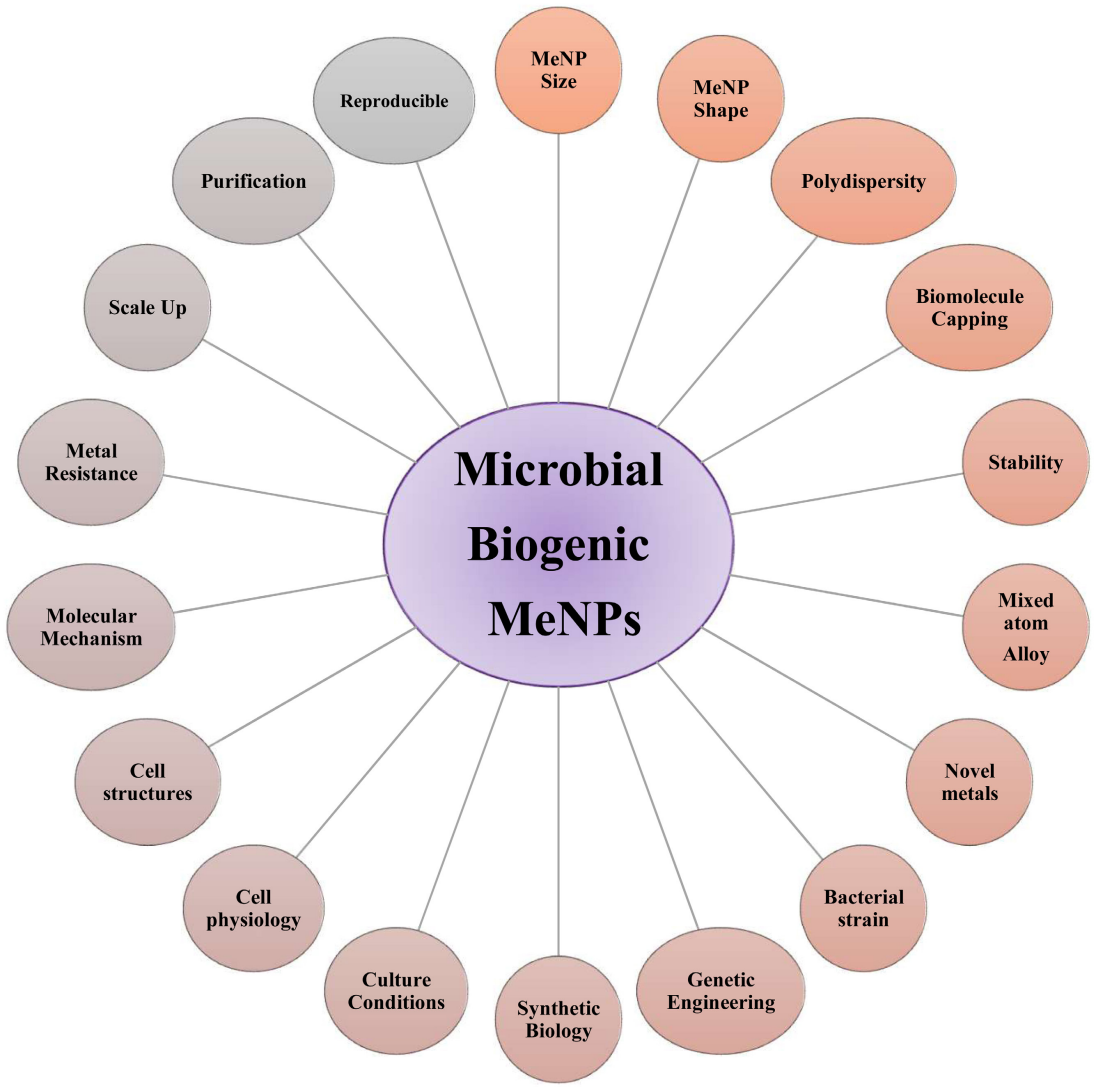
Microbial MeNP biotechnology: production variables.

Bacteria display an enormous potential as biofactories for MeNPs production since they convert metallic ions into MeNPs with a narrow size distribution and repetitive shapes. In addition, bacteria‐driven synthesis allows MeNPs capping with natural and biocompatible molecules that prevent aggregation and help in biomedical applications of the nanoparticles. Many bacterial species and strains have been described to date with good abilities to produce MeNPs (Gallo & Schillaci, 2021; Ghosh et al., 2021; Pradhan & Turner, 2022). Nevertheless, exploring new biofactories for MeNPs production is crucial to bypass some of the existing limitations (Figure 1). For example, some bacteria are able to produce SeNPs only under anoxic environments, where cells experience less oxidative stress and promote suitable environments for ionic reductions (Fernández‐Llamosas et al., 2016). Using fast‐growing bacteria is also a good feature since these biocatalysts would speed up production times. Thus, *Vibrio natriegens*, one of the fastest‐growth bacteria, is capable to synthesize significant amounts of SeNPs in only 6 h (Fernández‐Llamosas et al., 2017). Cyanobacteria and other photosynthetic bacteria are also attractive nanofactories for MeNPs production since the energy can be supplied by a removable source (solar light) and the carbon is captured as CO_2_, thus being a good example of a circular economy (Hamida et al., 2020). Additionally, extremophilic bacteria that are adapted to extreme environmental conditions (e.g., high metal concentration, high and low temperature, acidic or alkaline pH, high pressure, salinity and radiation) are particularly relevant to MeNPs biosynthesis (Atalah et al., 2022). Besides the harsh conditions that extremophiles can bypass, they also produce robust and stable capping proteins (Beeler & Singh, 2016), resulting in MeNPs of higher chemical stability (Correa‐Llantén et al., 2013). For instance, *Deinococcus radiodurans*, a radioresistant, thermotolerant, psychrotolerant and acid‐tolerant bacterium, has been shown to produce protein‐capped spherical silver NPs (Kulkarni et al., 2015). Another example is the thermophilic bacterium *Geobacillus wiegelii* strain GWE1, which produces SeNPs and TeNPs both intracellularly and extracellularly, and whose size and shape have been shown to be modulated by pH and temperature (Correa‐Llantén et al., 2014).

Although many metal ions interfere with the normal protein functions of bacteria and therefore are extremely toxic, a good number of metal hyper‐resistant bacteria have been already isolated. MeNPs can simply be produced by cultivating these bacteria that harbour molecules that interact with metals/metalloids, particularly metabolites, proteins or peptides involved in metal detoxification by mechanisms of in vivo reduction that are poorly understood (Park et al., 2015). Nevertheless, many metal hyper‐resistant bacteria have complex metabolic requirements, highly demanding growth conditions and are difficult to be genetically manipulated. As an alternative, one could engineer efficient MeNPs production using well‐known model bacteria that are sensible to metals but that can be endowed with genetic features to bypass some limitations such as resistance to toxic metals, an increase in speed of growth, use of simple growth media or production at high yields (Iravani & Varma, 2019, for a review; Figure 1). In Alonso‐Fernandes et al. (2023), one thiopurine methyltransferase from *Aromatoleum* sp. CIB was overproduced in *Escherichia coli* increasing its resistance to metalloids by more than three orders of magnitude. The modified bacteria speed up the elimination of tellurite/selenite from the medium and enhance the production of TeNPs/SeNPs.

Most bacteria display the production of MeNPs in the cytoplasm or periplasm (Thakkar et al., 2010). Therefore, another challenge is the production of MeNPs outside of the bacterial cell, thus avoiding extra costs of the breakdown of cells and complex protocols of purification from the cellular debris. In this sense, it has been shown that *E. coli* cells producing the SefA protein from *Thauera selenatis* are able to secrete SeNPs to the medium (Debieux et al., 2011).

## LESSONS FROM THE MOLECULAR DETERMINANTS RESPONSIBLE FOR MENPS PRODUCTION

Although some enzymes have been reported to play a role in metal/metalloid reduction and, hence, to be responsible for MeNPs synthesis in a few bacterial species, in most instances the molecular actors involved in MeNPs production have not been completely elucidated (Figure 1). It has been speculated that nitrite reductase, catalase, isocitrate dehydrogenase, lipoamide dehydrogenase, thioredoxin reductase, hydroperoxide reductase, NADH:flavorubredoxin or sulphite reductase are directly involved in Se‐ or TeNP production (Kessi et al., 2022 for a review). Reduced thiols are one the best candidates for bacterial intracellular metal/metalloid reduction (Kessi & Hanselmann, 2004). It has been also proposed that bacterial cell walls could act as nucleation sites for the synthesis of nanoparticle seeds, and for their further growth into MeNPs. Finally, it is well known that certain peptides, such as phytochelatin, or proteins, such as metallothionein, are overexpressed in microorganisms upon exposure to heavy metal ions (Cobbett & Goldsbrough, 2002). In summary, it appears that there are many different interconnected molecular mechanisms underlying MeNPs synthesis. The work of Avendaño et al. reported in this Special Issue provides insights into selenite metabolism in *Pseudomonas putida* KT2440. Their results suggest that the reduction of selenite occurs through an interconnected metabolic network involving central metabolic reactions, sulphur metabolism and the response to oxidative stress. Interestingly, genes such as *sucA*, *D2HGDH* and *PP_3148* revealed that the 2‐ketoglutarate and glutamate metabolism is important to convert selenite into selenium. In addition, mutations affecting the activity of the sulphite reductase decreased the bacteria's ability to transform selenite into Se(0). Interestingly, suppression of genes *sqrR*, *sqr* and *pdo2* resulted in the production of selenium nanoparticles at a higher rate than the wild‐type strain, which is an observation of great biotechnological interest (Avendaño et al., 2023).

## SEARCHING FOR THE OPTIMAL MENPS POLYDISPERSITY

The control of the characteristics and properties of nanoparticles, nanocomposites and nanoscale materials remain great challenge in the field of nanotechnology (Daryosush & Darvish, 2013). For instance, the activity of the SeNPs is size dependent, for example, the smallest SeNPs have the highest free radical scavenging potential (Torres et al., 2012). The polydispersity of biogenically produced MeNPs remains a big bottleneck, being mandatory to achieve stable systems to synthesize MeNPs of defined size and shape. Until now these two properties are constrained by the particular bacteria and growth conditions used in the production, making it difficult to design rational processes for the *à la carte* production of MeNPs (Fernández‐Llamosas et al., 2017). The formation of nanoparticles of various shapes and sizes can be controlled by changing the bioreduction conditions, including culture type and age, growth medium, culture liquid, cell extract, isolated proteins and incubation time. For example, biogenic SeNPs were represented exclusively by spheres whose size varied from 20 to 550 nm, depending on the culture conditions (Vetchinkina et al., 2019). The incubation time of the culture is crucial in SeNPs size production in *V. natriegens* (Fernández‐Llamosas et al., 2017) or in the length of the nanorods of tellurium in *Rhodococcus aetherivorans* BCP1 obtained in resting cells. The Te‐nanostructures initially appeared in the cytoplasm of BCP1 cells as spherical TeNPs, which were converted into nanorods at longer exposure times (Presentato et al., 2018). Bacterial MeNPs also show a variety of shapes with the typical structures of MeNPs nanoparticles synthesized by employing conventional chemical synthesis. For example, microbial reduction of gold ions produced octahedral, triangular, hexagonal and spherical shapes of gold nanoparticles (Park et al., 2015).

## CAPPING AND ITS RELEVANCE IN MENPS STABILITY

An important advantage of biogenic nanoparticles is their resistance to aggregation, which is ensured by the biological surface surrounding the MeNPs. This surface adsorbs some cellular components and biomolecules forming a corona that increases stability and biocompatibility (Monopoli et al., 2012). The capping layer has the ability to change MeNPs reactivity, structural integrity, thermodynamic and chemical stability and also influences functional properties. In fact, the monodispersity of biogenic SeNPs may reflect their natural stability within this size range due to the surrounding organic material produced by bacterial cells that control nanoparticle diameter (Debieux et al., 2011). These biomolecules are also responsible for the thermodynamic stability of biogenic SeNPs, as indicated by the formation of insoluble Se precipitates upon the physical removal of the organic material (Piacenza et al., 2019). The extraordinary importance of capping elements makes it essential to develop in the near future a data bank compiling the effect of cap‐associated biomolecules on the structural integrity and other related parameters of synthesized MeNPs (Bulgarini et al., 2021).

## FROM THE BENCH TO THE INDUSTRY: SCALING THE PROTOCOLS

Scaling the production yields from a laboratory to an industrial setup is always a complex process and a major challenge. In fact, while the biological synthesis of MeNPs has been achieved at a laboratory scale, only 1% of the microbial‐produced nanotechnology has been commercialized until now (Kapoor et al., 2021). In most cases, the yields of conversion of metal ions into biogenically produced MeNPs are either not investigated or poorly analysed. In fact, all the aspects concerning MeNPs production yields, including the time required in the process, must be conveniently investigated from the engineering point of view to achieve satisfactory industrial efficiencies. Despite the limitations on scaling up, there are some exploring on large‐scale synthesis of nanoparticles using biogenic routes with a narrow size distribution (Gahlawat & Choudhury, 2019). For instance, large‐scale production of magnetic and metal‐substituted magnetic nanoparticles using *Thermoanaerobacter* sp. TOR‐39 obtained at the end of the process about 1 kg of Zn‐substituted magnetites from 30 L fermentations similar quantities than achieved by traditional chemical synthesis. Factors such as biomass concentration, dosing amount, type of precursors used and the basal medium composition were found to be crucial for producing tailor‐made nanoparticles (Moon et al., 2010). More recently, the scalability of *Thermoanaerobacter* sp. TOR‐39 mediated ZnS nanoparticles production in 900 L scale bioreactors have been explored. The cultivation at 900 L scale yielded around 320 g ZnS nanoparticles powder (Moon et al., 2016).

## PERSPECTIVES ON THE SYNTHETIC BIOLOGY ERA

Synthetic and systems biology approaches are revolutionizing the perspective of biology and also the production of biomaterials by re‐engineering of life creating organisms capable of performing novel functions for industry, medicine and scientific research (Edmundson et al., 2014; Rice & Ruder, 2014). Engineer biological networks by remodelling genetic circuits or constructing new protein‐based components have been developed with promising impact on MeNPs production and some examples have been developed from the infancy of synthetic biology. For instance, the overproduction of two proteins, phytochelation and metallothionein in engineered *E. coli* allows the modified bacteria the production of several types of MeNPs including alkali earth, magnetic and semiconducting metals (Choi et al., 2018). The production of magnetic nanoparticles from magnetotactic bacteria in mammals (Kim et al., 2012) and yeast (Nishida & Silver, 2012) are also two examples of efforts to synthetically engineer magnetic nanoparticles in living cells. Linking the biorecovery of metals by bioconversion into MeNPs has been also explored using synthetic biology. A good number of bacteria with metal accumulation and recovery abilities will be good candidates for modification to enhance their abilities (Capeness & Horsfall, 2020). For example, a modular plasmid toolkit based on BioBrick assembly method was recently used in *Shewanella* to increase their ability of tungsten reduction (Cao et al., 2019).

Furthermore, since there are several MeNPs that have not yet been synthesized by chemical methods, and there are only a few studies on mixed MeNP production, such novel MeNPs can be potentially explored by bacterial synthesis and may serve as new nanomaterials for exciting industrial applications. Deeper knowledge of the molecular determinants involved in MeNPs synthesis and a wider number of bacterial chassis with abilities to produce MeNPs are required to transform engineered bacteria and enable them to serve as cellular bioreactors and nanofactories with the assistance of synthetic biology.

Besides the important progress achieved in microbe‐nanoparticle research in recent years and the limitations already discussed in this Editorial, further knowledge of the molecular mechanisms involved in the fermentation conditions applied will be essential to design rational and scalable processes to succeed at the industrial level (Figure 1).

## AUTHOR CONTRIBUTIONS

**Manuel Carmona:** Conceptualization (lead); writing – original draft (lead); writing – review and editing (lead). **Ignacio Poblete‐Castro:** Writing – original draft (supporting); writing – review and editing (supporting). **Mahendra Rai:** Writing – original draft (supporting); writing – review and editing (supporting). **Raymond J. Turner:** Writing – original draft (lead); writing – review and editing (lead).

## CONFLICT OF INTEREST STATEMENT

The authors declare no potential conflict of interest.
